# Orbital Abscess Developed Apart From Paranasal Sinusitis and Dacryocystitis in Fibrous Dysplasia

**DOI:** 10.7759/cureus.26061

**Published:** 2022-06-18

**Authors:** Yasuhiro Takahashi, Shinjiro Kono, Tatsuro Yokoyama, Tessei Kuruma, Aric Vaidya, Hirohiko Kakizaki

**Affiliations:** 1 Oculoplastic, Orbital & Lacrimal Surgery, Aichi Medical University Hospital, Aichi, JPN; 2 Otorhinolaryngology, Aichi Medical University, Aichi, JPN; 3 Oculoplastic, Orbital & Lacrimal Surgery, Kirtipur Eye Hospital, Kirtipur, NPL

**Keywords:** dacryocystitis, elevated intraocular pressure, sinusitis, orbital abscess, fibrous dysplasia

## Abstract

A 48-year-old man visited the emergency department of our hospital with swelling of the left upper and lower eyelids from the day before. On the first examination, he had severe swelling of the left upper and lower eyelids, proptosis, and chemosis. Left intraocular pressure was 33 mmHg. Computed tomographic images showed an orbital abscess in the anterosuperolateral orbital space, maxillary and ethmoidal sinusitis, and dacryocystitis. The orbital abscess was not contiguous to maxillary and ethmoidal sinusitis and dacryocystitis. Ground-glass appearance was seen in the frontal, maxillary, and ethmoid bones, and most of the space of the frontal sinus was obliterated due to the expansion of the frontal bone. Emergent drainage of orbital abscess, dacryocystorhinostomy, and endoscopic sinus surgery were performed under general anesthesia. Intravenous tazobactam/piperacillin was administered. A culture test of the sinus pus and orbital abscess showed growth of Streptococcus intermedius (2+). At one month postoperatively, there was no recurrence of orbital abscess, paranasal sinusitis, and dacryocystitis.

## Introduction

Fibrous dysplasia is a rare skeletal disorder characterized by fibrous replacement of the bone marrow [1]. This entity is not associated with ethnic and sex-related differences [2]. The common site of the involvement of the paranasal sinus skeleton is the sphenoid bone, followed by ethmoid and maxillary bones [1]. Fibrous dysplasia can obstruct the paranasal sinus ostium, causing acute paranasal sinusitis [3,4]. This rarely spreads directly into the orbit, resulting in orbital cellulitis and orbital abscess [3-6]. There had been only two reported cases of fibrous dysplasia with orbital abscess directly extended from paranasal sinusitis [3,4].

Here, we report a case of fibrous dysplasia with orbital abscess, which developed apart from paranasal sinusitis and dacryocystitis.

## Case presentation

This study was conducted in accordance with the tenets of the Declaration of Helsinki and its later amendments. Written informed consent for publication of an identifiable face photo was obtained from the patient.

A 48-year-old man presented to the emergency department of our hospital on a weekend night with swelling of the left upper and lower eyelids for one day. One month before his referral to us, he received antibiotics for paranasal sinusitis and orbital cellulitis for two weeks at another hospital. He was clinically diagnosed with fibrous dysplasia previously. He had no history of any immunocompromising disease or facial trauma.

**Figure 1:**
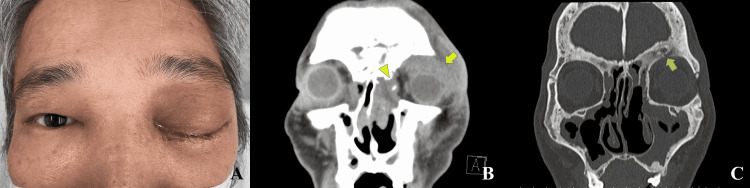
Case presentation (A) A face photo was taken at the first examination showing upper and lower eyelids swelling, proptosis, and chemosis on the left side. (B, C) Coronal computed tomographic images showing orbital abscess (yellow arrow), maxillary and ethmoidal sinusitis, and dacryocystitis (yellow arrowhead). Ground-glass appearance is seen in the frontal, maxillary, and ethmoid bones, and most space of the frontal sinus is obliterated. Cystic lesions are seen in the frontal and maxillary bones, and one cyst opens toward the superior orbit (green arrow).

On the first examination at the emergency room, there was difficulty in opening the left eye due to severe left upper and lower eyelids swelling and proptosis (Figure 1A). Light reflex was prompt in both eyes. The left eye was positioned in the inferior gaze, and it could not move in the superior direction. The left intraocular pressure measured using iCARE® (Vantaa, Finland) was 33 mmHg. Slit-lamp examination revealed severe chemosis in the left eye. Computed tomography (CT) images showed an orbital abscess in the anterosuperolateral orbital space, maxillary and ethmoidal sinusitis, and dacryocystitis (Figures 1B, 1C). An orbital abscess developed apart from maxillary and ethmoidal sinusitis and dacryocystitis. Ground-glass appearance was seen in the frontal, maxillary, ethmoid, and zygomatic bones, which corresponded to the previous diagnosis of fibrous dysplasia. Most of the space of the frontal sinus was obliterated due to the expansion of the frontal bone. Small cystic changes were demonstrated in the frontal and maxillary bones, and one cyst opened toward the superior orbit. The lacrimal sac fossa had a partial defect. A blood test revealed a high white blood cell count (10,600/μl) and elevated C-reactive protein (14.50 mg/dL).

After hospital admission, emergent drainage of the orbital abscess, dacryocystorhinostomy, and endoscopic sinus surgery were performed under general anesthesia. Polyps in the middle nasal meatus were removed, and the pus was drained. A thickened uncinate process and ethmoid sinus septa were removed using a drill with a diamond burr to open the posterior ethmoid sinus and superior nasal meatus. The lacrimal sac was opened, and a lacrimal tube was inserted. A sub-brow incision and lateral canthotomy along with cantholysis were performed to drain the orbital abscess. We confirmed no connection between the superolateral orbital space and paranasal sinus. A drain was placed in the superolateral orbital space. The lateral canthus was left unsutured to keep the intraocular pressure reduced.

Intravenous tazobactam/piperacillin was administered, and the orbital space was irrigated from the drain. The results of the culture test of the pus, obtained at five days postoperatively, showed growth of *Streptococcus intermedius* (2+). As this microorganism was found to have high drug sensitivity for tazobactam/piperacillin, we continued the antibiotic, as well as the irrigation, till the ninth postoperative day. As *S. intermedius* has been isolated from patients with periodontitis, the patient was consulted with a dentist. However, the relationship between the intraoral condition and paranasal/orbital infection was unclear. At nine days after surgery, the lateral canthus was sutured and re-fixed, and at 13 days after surgery, the patient was discharged from the hospital.

At one month postoperatively, there was no recurrence of orbital abscess, paranasal sinusitis, and dacryocystitis. Intraocular pressure decreased to 19 mmHg. The vision was normal and the extraocular muscle motility improved.

## Discussion

We report a patient with fibrous dysplasia who showed orbital abscess, maxillary and ethmoidal sinusitis, and dacryocystitis. There was no direct connection between the orbital abscess and paranasal sinusitis. Although dacryocystitis can cause orbital abscess [7], the orbital abscess was far away from the dacryocystitis in this case. There had been only two reported cases of fibrous dysplasia with orbital abscess, which was caused by the direct spread of paranasal sinusitis [3,5]. A possible etiology in this study was an indirect hematogenous spread of ethmoidal sinusitis into the anterosuperolateral orbital space [4]. Another possible etiology was the secondary transformation of aneurysmal bone cysts in the frontal bone shown as cystic lesions on CT [3], although a biopsy of the frontal bone was not performed. One cyst opening toward the superior orbit allowed the accumulation of orbital hematoma [3], which might have been an infection source in this case.

Urgent drainage of the orbital abscess is required to prevent the development of serious complications, including visual loss and other lethal conditions, such as cavernous sinus thrombosis, meningitis, and cerebral abscess [8]. Also, broad-spectrum intravenous antibiotics should be given until obtaining the results of cultural tests [8]. Our treatment plan followed this standard treatment regimen. Furthermore, the lateral canthal ligament was left disinserted to reduce both the intraocular and retrobulbar pressures in this case [9].

## Conclusions

In conclusion, we report a rare case of fibrous dysplasia and orbital abscess with no contiguous spread of paranasal sinusitis. Indirect hematogenous spread of ethmoidal sinusitis and secondary transformation of aneurysmal bone cysts in the frontal bone may be possible etiologies of the orbital abscess. Urgent surgical and medical treatments are necessary to prevent the development of serious complications.
